# A Conversation at Charing Cross Hospital?

**Published:** 1915-08-07

**Authors:** 


### THE BRITISH HOSPITALS ASSOCIATION.

### A Conversation at Charing Cross Hospital?

The attendance at the meeting of the British Hospitals Association at Charing Cross Hospital on the 30th ultimo, under the presidency of Mr. H. Wade Deacon, Chairman of the Liverpool Royal Infirmary, was disappointing. Those present were: the representatives of St. Bartholomew's, Charing Cross, Royal Free, Seamen's, and Westminster Hospitals. Other hospitals represented were: the Royal Infirmary, Liverpool; Newport Hospital, Mon.; General Hospital, Northampton; Gravesend, Pontypool, Wellingborough, and Wolverhampton Hospitals. There were also two representatives from the British Medical Association, Sir William J. Collins and Mr. H. R. Maynard (Secretary of King Edward's Hospital Fund).

The subject for discussion was the means of meeting the difficulty which had arisen in voluntary hospitals due to the shortage of medical officers arising out of the war. During recent months this difficulty, we have been able to report, has been largely solved by the greater of the voluntary hospitals, and it is hoped that by a readjustment of the system and inter-communication between individual hospitals and the central authorities and organisations, outstanding difficulties can be speedily reduced to a minimum.

The discussion soon revealed that the meeting of the British Hospitals Association, to prove effective from its point of view, should have been held much earlier in the year. At the commencement the speeches consisted mainly of a summary of the steps already taken to solve the difficulties, and the practical value of the discussion was confined to a reference to the systems introduced at St. Bartholomew's, the London, and some other hospitals; the proposed action of the General Medical Council with a view to the utilisation of practitioners possessing Canadian and other Colonial qualifications; and to information given by the representatives of the British Medical Association.

It was stated that at twenty-six London hospitals which in July 1914 had 104 resident medical officers, there were now only eighty-one.

